# Surface Electromyography Monitoring of Muscle Changes in Male Basketball Players During Isotonic Training

**DOI:** 10.3390/s25051355

**Published:** 2025-02-22

**Authors:** Ziyang Li, Bowen Zhang, Hong Wang, Mohamed Amin Gouda

**Affiliations:** 1 Department of Mechanical Engineering and Automation, Northeastern University, Wenhua Street, Shenyang 110819, China; 2 Physical Education Department, Northeastern University, Wenhua Street, Shenyang 110819, China

**Keywords:** electromyography, feature extraction, resistance training, muscle changes, machine learning

## Abstract

Physiological indicators are increasingly employed in sports training. However, studies on surface electromyography (sEMG) primarily focus on the analysis of isometric contraction. Research on sEMG related to isotonic contraction, which is more relevant to athletic performance, remains relatively limited. This paper examines the changes in the isotonic contraction performance of the male upper arm muscles resulting from long-term basketball training using the sEMG metrics. We recruited basketball physical education (B-PE) and non-PE majors to conduct a controlled isotonic contraction experiment to collect and analyze sEMG signals. The sample entropy event detection method was utilized to extract the epochs of active segments of data. Subsequently, statistical analysis methods were applied to extract the key sEMG time domain (TD) and frequency domain (FD) features of isotonic contraction that can differentiate between professional and amateur athletes. Machine learning methods were employed to perform ten-fold cross-validation and repeated experiments to verify the effectiveness of the features across the different groups. This paper investigates the key features and channels of interest for categorizing male participants from non-PE and B-PE backgrounds. The experimental results show that the F12B feature group consistently achieved an accuracy of between 80% and 90% with the SVM2 model, balancing both accuracy and efficiency, which can serve as evaluation indices for isotonic contraction performance of upper limb muscles during basketball training. This has practical significance for monitoring isotonic sEMG features in sports and training, as well as for providing individualized training regimens.

## 1. Introduction

Resistance training is a type of exercise that works against external resistance, primarily designed to enhance muscular strength. This training enhances upper body explosive strength in young basketball players [1]. It has become a fundamental element of every athlete’s conditioning program [2]. Traditional resistance training includes exercises such as push-ups, exercises using dumbbells and barbells, and other activities. The benefits of this training include delaying the effects of aging, decreasing body fat percentage [3], mitigating injury risk and alleviating pain, changing body shape, improving posture, and increasing bone density. Neuromuscular activation can be classified into isotonic, isometric, and isokinetic muscle contractions [4] under different movement patterns, physiological states, tasks, and training regimes. Isotonic contractions have a more significant impact on athletic performance [5] because they involve dynamic movement and adaptive changes. In contrast, isometric contractions are mainly utilized in strength training and rehabilitation. Jumanza et al. [6] investigated the impact of muscle strength indices on shooting performance during isometric training.

Experts in the field of traditional physical training are more adept at utilizing meta-analysis methods. Schoenfeld et al. [7] described the effects of total weekly resistance training volume on muscle mass changes through meta-regression. Nuzzo [8] reviewed sex differences in numerous strength training variables and outcomes. With the advancement of computer technology, analysis methods based on physiological signals, such as electroencephalogram (EEG), electrocardiogram (ECG) [9], and electromyography (EMG) analysis, have significantly matured. Wang et al. [10] explored inter-athlete trust behaviors and their neural mechanisms with fNIRS technology. Compared to EEG analysis, EMG is currently closer to practical applications, such as recognizing movement intent [11], controlling exoskeletons [12], assessing muscle fatigue [13], and developing rehabilitation robots [14]. Gesture recognition [15] is another area of interest. Especially in the field of sports, sEMG (surface electromyography) signals analysis has been employed to enhance the competitive level of athletes. Barnamehei et al. [16] focused on musculoskeletal biomechanics simulation of badminton athletes, and Smerdov et al. [17] researched eSports players. To improve sports performance, repetitive training is essential for developing muscle memory [18], just as repetitive learning can deepen knowledge retention. However, most studies on EMG-based muscle assessment are conducted under isometric contractions, which cannot fully capture the characteristics of muscle changes during dynamic movements [19].

To the best of our knowledge, EMG research on isotonic contractions is limited, although it offers insights into various aspects of muscle function, including muscle activation, motor unit recruitment, muscle fatigue [20,21,22], rehabilitation, and injury prevention. The dumbbell curl, a common resistance training exercise, induces isotonic muscle contractions, making it an easily implementable experimental method for related research. Karthick et al. [23] instructed subjects to perform continuous bicep curl exercises using only their dominant hand to lift a dumbbell, while Reeves and McLean [24] instructed participants to hold a weight statically with one arm while performing repeated dynamic elbow flexion and extension contractions with the other arm. However, both studies primarily focused on analyzing muscle fatigue.

This study aims to explore the minimal combinations of features sufficient for monitoring upper muscle changes during isotonic contractions in the training process of male basketball players. It is important to investigate whether there are general sEMG features across different muscle groups rather than focusing solely on the specific muscles of B-PE and non-PE subjects. We consider B-PE and non-PE participants as representing two distinct levels of muscle training. The goal is to identify more universally applicable sEMG features as indicative metrics of muscle performance [25] during isotonic contractions, such as absolute strength, endurance, current and potential ability, etc. These findings can provide more precise strategies and adaptations for individuals’ training programs by longitudinally monitoring changes in metrics throughout the basketball training process. While this paper focuses on distinguishing EMG characteristics between two distinct groups, the results can aid sports specialists in conducting more nuanced studies on training levels and phases.

**Figure 1:**
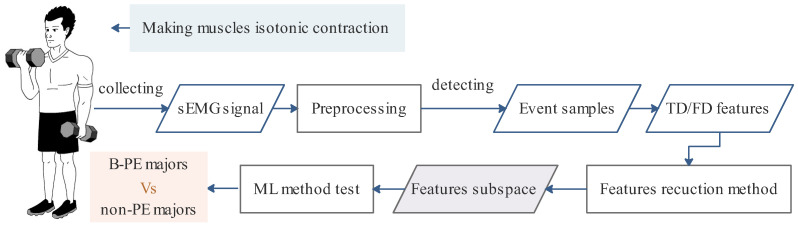
The framework of this paper.

As shown in Figure 1, this study attempts to propose an applicable method for universally evaluating muscle training effectiveness under isotonic contraction by extracting sEMG features using statistical analysis methods. The feature subspace includes key TD and FD features of arm muscles during isometric movements. The ML method based on the feature subspace can distinguish B-PE and non-PE students, suggesting that extracting the feature subspace will be affected by basketball training. The redundancy of feature vectors is reduced, and the effectiveness of feature vectors is validated using a machine learning (ML) classifier. The results can be applied to the graded physical teaching and training for athletes, which provides a reference for the study of the universal sEMG characteristics across multiple muscle groups during isotonic contraction exercises.

## 2. Material and Methods

### 2.1. sEMG Acquisition

At Northeastern University, students from the Physical Education Department were recruited as the professional basketball group, while those from the Department of Mechanical Engineering formed the amateur group. These two groups represent different levels of upper body muscle training. In the end, five male participants were recruited for each of the two groups, and their relevant information is presented in Table 1. The B-PE students participated in significantly more weekly exercise than their mechanical engineering counterparts.

**Figure 2:**
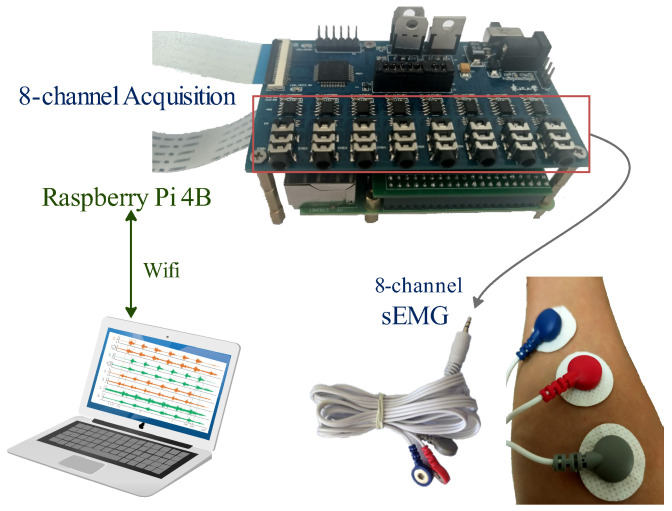
Hardware system for sEMG acquisition.

Tripolar electrodes were used to collect differential voltage signals from sEMG. The arrangement of the button electrodes and preamplifier [26] is illustrated in Figure 2. We used disposable electrodes to obtain high-quality signals. During dynamic conditions, sweat and skin movement can compromise electrode adhesion, typically degrading signal quality within 1 to 3 h. The three electrodes were arranged in blue, red, and gray order, each with a diameter of 25 mm and a buckle size of 3.9 mm. The homemade preamp [11,27], multi-channel analog acquisition supports up to 8 channels of sEMG, which can eliminate some signal artifacts and amplifies the original EMG voltage value by 2000 times. Eight sEMG adhesive button electrodes, along with their data cable (ZTEMG-1300, ZhiTuo, Qingdao, China), were connected to the 16-bit precision analog-to-digital converter (AD7606) on the amplifier circuit board. The Raspberry Pi 4B served as the main control board, receiving data via an I²C interface from the preamplifier. The data were then transmitted to a PC via WiFi for subsequent analysis with a sampling frequency of 1000 Hz.

### 2.2. Experimental Procedure

**Figure 3:**
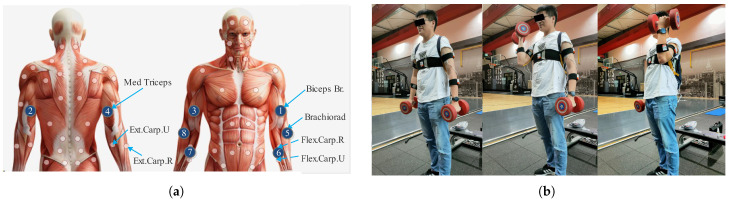
Data collection implementation program: target muscles to be detected (**a**), and standing alternating dumbbell curl (**b**). Sensors 1, 2, 5, and 6 are positioned on the biceps, triceps, brachioradialis, and flexor carpi muscles of the left arm, respectively. Symmetrically, sensors 3, 4, 8, and 7 are placed in the corresponding locations on the right arm in the same order.

Given the arm muscles involved in a standing dumbbell alternating curl, eight muscle regions were selected for sEMG signal acquisition, including the biceps, triceps, and the radial and ulnar wrist flexor/extensor muscle groups (Flex. Carp. U and Ext. Carp. U) of both arms. In Figure 3a, the serial numbers indicate the sequence of eight acquisition channels.

The experiment followed the European SENIAM standards [28], which provide recommendations on sensors, sensor placement, signal processing, and modeling [29]. Before the experiment, participants warmed up for two minutes to minimize injury risk, and the electrode sites were cleaned with alcohol wipes before electrode placement at the eight positions shown in Figure 3a. The placement of the electrodes adhered to SENIAM standards, with a center-to-center distance of approximately 20 mm. The sEMG acquisition channels were arranged according to the sequence of target muscles shown in Figure 3a.

We asked participants to perform standing alternating dumbbell curls after completing the tutorial by [30] using a 5 kg dumbbell in each hand, as shown in Figure 3b. Each trial consisted of 1 s of flexion and 1 s of extension per arm, alternating for six cycles. Thus, completing a single-arm curl needed about 2 s, while an alternating curl required approximately 4–5 s, making a total of about 30 s for six repetitions. To minimize the impact on the EMG signal, all subjects were asked move at approximately the same speed each time. Electrodes were checked at the end of each trial and replaced if loose; the trial was repeated if necessary. The sequence of using the left and right arms was based on individual preference and not strictly enforced.

Each subject conducted five trials, with a 1 min interval between trials. Adequate rest between trials is essential, because resistance exercise primarily relies on anaerobic metabolism, causing the muscles to reach fatigue relatively quickly. Short rest intervals (i.e., 30 to 90 s) are typically recommended for hypertrophy and muscular endurance training [2]. In total, 50 trials were conducted, yielding 5 × 10 datasets. After data preprocessing and event detection, 8-channel signals were obtained for each curl. Finally, statistical analysis and ML methods were applied to classify B-PE and non-PE participants based on the key EMG features, assessing whether these metrics effectively distinguish different upper limb training levels.

### 2.3. sEMG Signals Analysis

#### 2.3.1. Data Preprocessing

**Figure 4:**
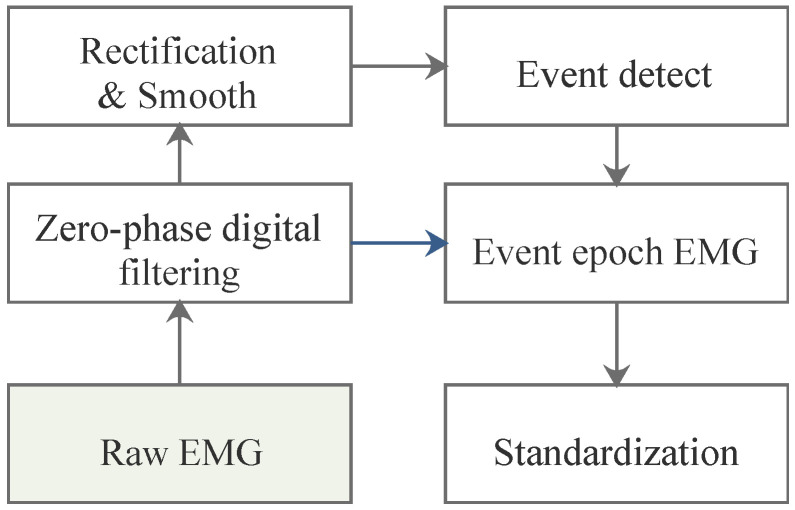
sEMG signals preprocessing and event detection.

The complete procedure for preprocessing and event detection is illustrated in Figure 4. First, all the raw signals were filtered to reduce noise. Simultaneously, events were detected to identify the starting and ending points of each arm curl motion, which were defined as active epochs. We then extracted all epochs from filtered data. To enhance event detection results, we applied rectification and smoothing processes. Without these steps, the extracted EMG signals from the detected event epochs would have lost valuable feature information. Finally, all activity segment samples from 80 sets (grouped by 10 subjects across 8 channels) were standardized separately.

**Figure 5:**
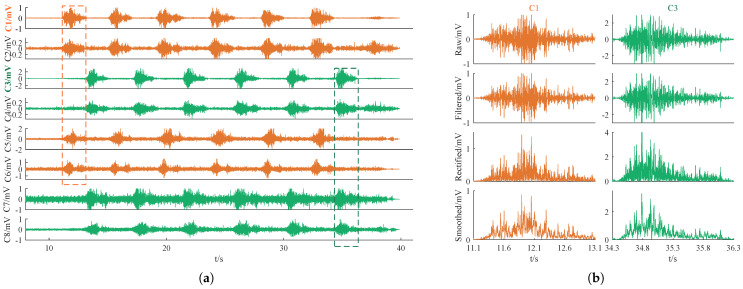
Raw 8-channel sEMG signals in one trial (**a**), and the sketch map of signal preprocessing (**b**).

As an example, the 8-channel raw data are shown in Figure 5a. C1–C8 correspond to the patch positions in Figure 3a, where C1 and C3 represent the positions of the biceps brachii muscles in the left and right arms, respectively. In this example, we can see that the left arm moves first and the right arm completes the motion last. Note that, for better visualization, the amplitude coordinate scale of each channel is not consistent here. The goal of the preprocessing stage is to extract the sEMG signals of each active epoch, as indicated by the first and last dashed rectangular box in Figure 5a, which correspond to the first epoch of the left arm and the last epoch of the right arm, respectively. We performed event detection on each trial, dividing it into two parts based on the left and right arms, each with four channels of sEMG signals. The biceps brachii signals of each arm were selected for active segment detection and the output active duration was applied to the other three channels. Specifically, the detection results of the C1 active segment enabled the extraction of active epoch signals from the left arm’s four channels (C1, C2, C5, and C6). Similarly, C3 corresponds to the four channels (C3, C4, C7, and C8) of the right arm. Ultimately, data were obtained for all curl segments, yielding 30 epochs per channel per subject. This totaled 300 epochs in each channel, with amateur and professional participants accounting for half of the data each.

Firstly, a zero-phase filter [31], specifically, a 4th-order 20–250 Hz bandpass Butterworth filter, was applied to remove baseline drift and measurement noise from the raw data. Since power frequency notch filtering has already been performed in the analog circuits, tests have confirmed that repeatedly conducting digital power frequency notch filtering can lead to excessive loss of useful information, adversely affecting subsequent event detection and feature extraction. Therefore, we chose not to perform power frequency bandstop filtering in this case. To extract the active epochs in the filtered signals, half-wave rectification and moving average smoothing with a span of 5 were applied. This process smoothed the signal, facilitating the next step in sample entropy-based active segment detection. It is important to note that the rectification and smoothing were solely for event detection; the final event epoch data used for feature analysis were extracted from the filtered signal, which retained 50% of the energy. Taking the C1 and C3 data segments intercepted from Figure 5a as an example, the effects of the three-stage preprocessing are illustrated in Figure 5b. Note that the signals from the other six channels were only filtered, without undergoing rectification.

**Figure 6:**
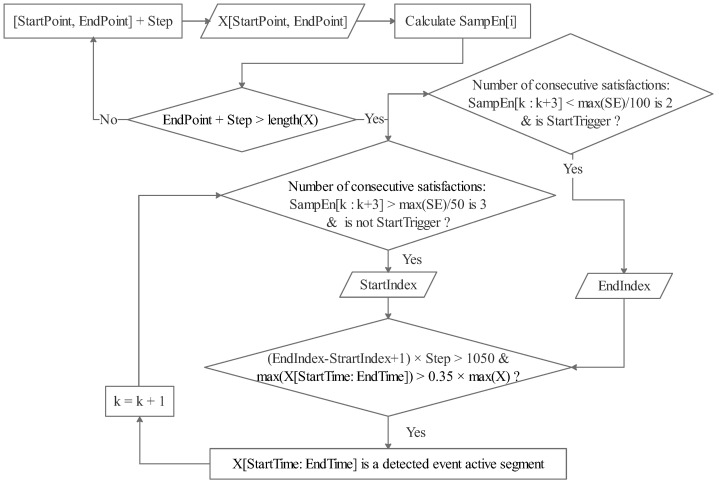
Flow chart of event detection by sample entropy.

Sample entropy (SampEn) demonstrates strong performance in event detection [32]. It effectively characterizes the continuity of intermittent signals and offers a broader range for threshold selection. Additionally, it can accurately represent electromyographic signals with smaller amplitudes. Richman and Moorman [33] introduced the mathematical definitions of ApEn(m,r,N) and SampEn(m,r,N), comparing their effectiveness. Here, the key parameters m,r,N represent the length of the sequences to be compared, the tolerance for accepting matches, and the length of all sample points, respectively. The event detection method based on sample entropy involved two steps: calculating the SampEn value sequence and then making conditional judgment for event epochs, as illustrated in Figure 6. In the first step, we preset m=2,r=0.25∗SD(X), where *X* represents the sample sequences, making *r* a global tolerance. A rectangular window of width 64 was iterated in steps of 30 to obtain data and calculate the SampEn of *X*. In the second step, we set a threshold factor of 0.02 for the start point and 0.01 for the end point, along with anti-shake criteria: 3 for the start mark and 2 for the end mark. To filter out incorrect results, the event epoch had to satisfy specific conditions, including a length greater than 1050 and a maximum amplitude exceeding 0.35∗X. All parameters were empirically determined based on testing.

**Figure 7:**
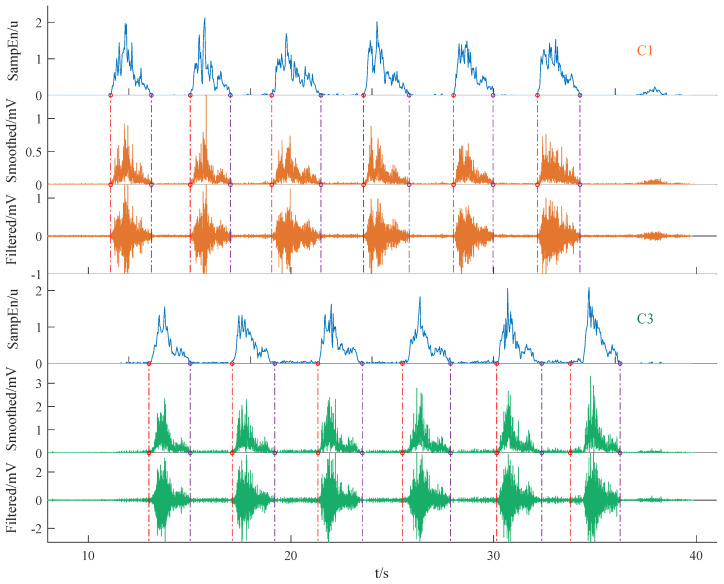
Event epoch signal interception. Here, u shows that sample entropy is unitless.

The event detection results are shown in Figure 7, where the sample entropy of the C1 and C3 signals determines the start and end points of the extracted active segment data. SampEn was computed using smoothed signals, whereas filtered data were used to extract active segments, thereby preserving more signal features. Specifically, we filtered the signals of all channels and ultimately extracted event epochs from the filtered data based on the event detection results from C1 and C3. The extracted event epoch typically exhibited a high peak followed by a low peak, resembling a tadpole with a small protrusion at its tail when we zoomed in along the horizontal axis, as shown in Figure 5b.

(1) $$NEMG=EMG_{i}/max\left(\left|EMG_{all}\right|\right)$$

Finally, to eliminate individual differences and account for the maximum amplitude variations among different muscle groups within the same subject, all trial samples $EMG_{all}$ per channel per participant were standardized (see Equation (1)). Here, $EMG_{i}$ represents a single sample point within all event epochs extracted from the signal after applying a bandpass filter to a specific channel for a given subject. The following section focuses on feature analysis using the standardized NEMG data.

#### 2.3.2. Feature Extraction

Feature extraction is crucial in sEMG signal applications as it directly affects functional reliability. Common features of digital signals include TD, FD, and time-frequency domain (TFD) features [34]. Phinyomark et al. [35] summarized thirty-seven time domain and frequency domain features. Time-frequency methods have been utilized for fatigue assessment [23] and for classifying hand movements [36]. Nazmi et al. [37] employed reverse arrangement (RA) and modified reverse arrangement (MRA) tests to determine whether EMG signals meet stationarity conditions. S. et al. [38] employed geometric features of sEMG signals combined with the discrete Fourier transform (DFT).

Based on a literature review, we selected 50 commonly used features in total, consisting of 41 TD features [39] and 9 FD features [35], calculated using a MATLAB M-script file. The 41 TD features were ASM, ASS, AR, AAC, AE, CARD, COV, DAMV, DASDV, DVARV, EMAV, EWL, IEMG, IQR, KURT, LCOV, LD, LDMA, LDASDA, LTKEO, MFL, MAS, MAV, MSR, MMAV, MMAV2, MYOP, FZC, RMS, SSI, Skew, SSC, SD, TM, VAR, VARe, VO, WL, WA, ZC, and DUR. The 9 FD features were MDF, MNF, FR, PKF, PSR, MNP, SM, VCF, and WP. Each feature is indexed as f+(feature index number) for reference. In the subsequent sections, we use F+(number of features) to denote the selected feature space. Because the 4th-order AR coefficients consist of 4 values, SM includes the first four spectral moments (SM0–SM2). Additionally, wavelet packet decomposition at level 3 yields 8 values, resulting in a total of 63 feature values for an event epoch. The feature space consists of 300 trials × 63 feature values × 8 channels, with all 300 trials being standardized for both amateurs and professionals per feature per channel.

**Figure 8:**
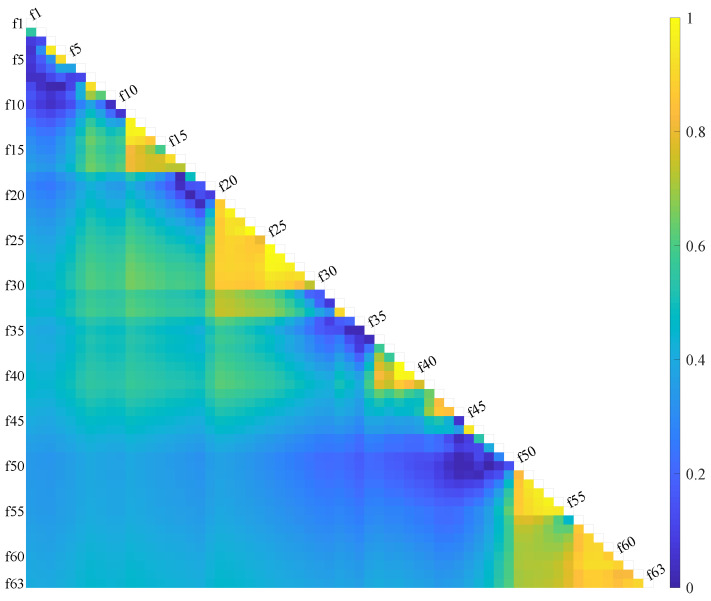
Consistency and correlation between every two features. The color means the intraclass correlation coefficient. fx means the x-th feature. The closer it is to yellow, the higher the consistency, and blue means non-consistency. We believe that the performance of two features with coefficients greater than 0.8 is consistent, as with [f7, f8], [f11, f12, f13, f14]; [f15, f16]; [f20~f30]; [f32, f33]; [f38~f41]; [f42~f44]; [f51~f55]; and [f57~f63]. All of these are feature groups with significant correlation that can reduce redundancy.

Given the large number of features, consistency and correlation analyses were performed for feature reduction and selection. The intraclass correlation coefficient (ICC) is an index for assessing the agreement of measurements taken by different observers for quantitative responses [40] and is widely applied in the biomedical field [41]. We computed the correlations among the 63 features by averaging the feature space across the 8 channels and applying a one-way ICC approach. In Figure 8, the yellow areas indicate higher consistency.

**Figure 9:**
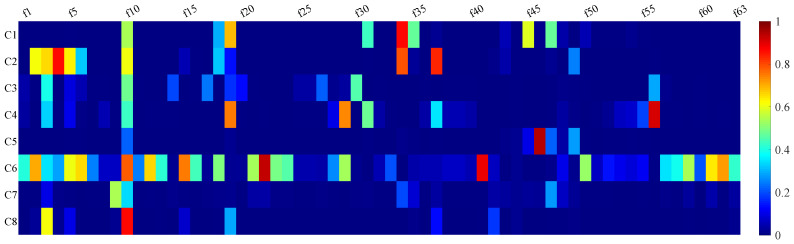
Two-sample t-test for amateurs and professionals with 63 features in 8 channels. The color means the *p*-value of two-sample *t*-test at the default 5% significance level.

Additionally, we examined the differences between the two groups (B-PE and non-PE) using a two-sample t-test at a significance level of 0.05. The resulting p-value matrix is presented in Figure 9, and the H-value indicates the test decision regarding the null hypothesis. Notably, the C6 channel signals exhibited relatively lower significance in differentiating between B-PE and non-PE students compared to other channels. This might be due to experimental variability, and more samples are needed for further investigation. To enhance the accuracy of key feature identification, excluding the C6 channel and relying on the other seven channels for feature analysis and selection may be beneficial. Thus, we employed two methods (Methods A and B in Appendix A) for feature reduction.

The key difference between Method B and Method A is that channel 6 was excluded when determining the conditions for *p*-values and *H*-values, introducing an extra step (step 0). In step 1, we identified specific features indexed within $Z_{1}$ for removal, resulting in feature combinations named F50A and F50B, respectively. In step 2, we utilized the feature correlation results shown in Figure 8 to further reduce the dimensionality of the remaining features, yielding feature combinations indexed within $Z_{2}$ named F18A and F16B, respectively.

In order to further reduce the computational cost, additional features indexed within $Z_{3}$ were removed by applying conditions in step 3, resulting in feature combinations named F12A and F12B, respectively. The feature index order corresponds to the 63 feature values listed in Section 2.3.2. Ultimately, F12A consists of feature indices [1, 8, 14, 17, 25, 32, 36, 40, 44, 48, 51, 60], while F12B is indexed with [1, 6, 7, 11, 23, 33, 36, 41, 44, 48, 51, 60]. To verify the effectiveness of the extracted features, we introduced a control group, F12X, derived from F12A by replacing F8 and f14 with F78 and F11 in step 2 and substituting F17 with f9 in step 3.

The selected 16 features from Method B (F16B) are explained and defined as follows:

1. ASM: Absolute value of the summation of the expth root and its mean, which gives an approximate measure of the power of the signal [42], defined as
  (2) $$\begin{array}{cc} \; & ASM=\left|\frac{\sum_{n=1}^{k}{\left(x_{i}\right)}^{exp}}{N}\right|,\\ \; & exp=\left\{\begin{array}{c} 0.5,\;if(i\geq 0.25*N\;\&\;i\leq 0.75*N)\\ 0.75,\;otherwise \end{array}\right. \end{array}$$
  where *N* is the length of *X*.
2. AR(4) and AR(2): the 4th and 2nd coefficient of autoregressive parameters corresponding to a model of order 4 [35], which is
  (3) $$y\left(n\right)=\sum_{k=1}^{4}a(k)*y(n-k)=x\left(n\right)$$
3. AAC: Average Amplitude Change [35]:
  (4) $$AAC=\frac{1}{N}\sum_{i=1}^{N-1}\left|x_{i+1}-x_{i}\right|$$
4. DAMV: Difference Absolute Mean Value [35]:
  (5) $$DAMV=\frac{1}{N-1}\sum_{i=1}^{N-1}\left|x_{i+1}-x_{i}\right|$$
5. LTKEO: Log Teager–Kaiser Energy Operator [43]:
  (6) $$LTKEO=\ln\sum_{i=2}^{N-1}\left({x_{i}}^{2}-x_{i-1}*x_{i+1}\right)$$
6. SSI: Simple Square Integral [35]:
  (7) $$SSI=\sum_{i=1}^{N}{x_{i}}^{2}$$
7. SD: Standard deviation [44]:
  (8) $$\begin{array}{cc} \; & SD=\sqrt{\frac{1}{N-1}\sum_{i=1}^{N}{\left|x_{i}-\mu \right|}^{2}}, \end{array}$$
  where $\mu =\frac{1}{N}\sum_{i=1}^{N}x_{i}$.
8. WL: Wave Length [45], which can be calculated by simplifying the cumulative length of the waveform summation:
  (9) $$WL=\sum_{i=1}^{N}\left|x_{i}-x_{i-1}\right|$$
9. DUR: The duration (customized metrics) of an event epoch extracted by SampEn method is defined as follows:
  (10) $$\begin{array}{cc} \; & DUR=\frac{N}{F_{s}}, \end{array}$$
  where $F_{s}$ is the sample frequency, 1000 Hz.
10. PKF: Peak Frequency at which the maximum power occurs [35]:
  (11) $$PKF=f\;where\;P_{f}=max\left(P_{j}\right),$$
  where $P_{j}$ is the EMG power spectrum at frequency bin $j,\;j=1,2,\cdots ,F_{s}/2$.
11. SM0: Zero Spectral Moment, also named total power (TTP) [35], which is defined as an aggregate of the EMG power spectrum. The k-th moment (SMk) is
  (12) $$SMk=\sum_{j=1}^{F_{s}/2}P_{j}*{f_{j}}^{k},$$
  where $f_{j}$ is the frequency of the spectrum at frequency bin *j*.
12. Wprcoef(34): Reconstruct wavelet packet coefficients [46] firstly decompose the signal at level 3 with db1 wavelet packets using Shannon entropy, then reconstruct the packet at node (3, 4) and calculate the Euclidean norm of vector $v_{k}$:
  (13) $$∥v∥=\sqrt{\sum_{k=1}^{L}{\left|v_{k}\right|}^{2}},$$
13. ASS: Absolute value of the summation of the square root [42]:
  (14) $$ASS=\left|\sum_{n=1}^{k}{\left(x_{n}\right)}^{1/2}\right|$$
  where *k* represents the analysis window, and $x_{n}$ denote the data within the corresponding analysis window.
14. CARD: Cardinality [45] is defined as follows:
  (15) $$\begin{array}{cc} \; & CARD=\sum_{i=1}^{N-1}Z_{i}\\ \; & Z_{i}=\left\{\begin{array}{c} 1,\;if\left|x_{i+1}-x_{i}\right|>0.01\\ 0,\;otherwise \end{array}\right. \end{array}$$
  where X has been randomly shuffled in order.
15. MNF: Mean Frequency [35], an average frequency which is calculated as the sum of the product of the EMG power spectrum and the frequency divided by the total sum of the spectrum intensity:
  (16) $$\begin{array}{cc} \; & MNF=\frac{\sum_{j=1}F_{s}/2f_{j}*P_{j}}{\sum_{j=1}F_{s}/2P_{j}}, \end{array}$$
  where $j=1,2,\cdots ,F_{s}/2$.

## 3. Results

### 3.1. Performance of Metrics in Categorization

Classification models were trained using four machine learning methods: the Linear Discriminant Analysis (LDA), Support Vector Machine (SVM), kernel-based SVM (SVMcore), and Subspace Discriminant (Ensemble) methods. For each training iteration, we randomly stratified the samples by category, shuffled the overall order, and applied ten-fold cross-validation. The classification accuracy was calculated using Equation (17), where higher accuracy indicates a better classifier. Each machine learning method was evaluated over twenty iterations to obtain the mean and variance of accuracy, as presented in Table 2. We examined three feature combinations with a dimension size of 12 named F12X, F12A, and F12B. Considering both mean accuracy and standard deviation—mean and standard deviation—the F12B feature combination exhibited the best performance in the SVM2 model. Although the specific accuracy values varied across the four ML methods, the overall trend was consistent across channels, with the C6 channel exhibiting relatively lower accuracy.

(17) $$accuracy=\frac{1}{k}\sum_{k=1}^{K}{\left[\frac{TP+TN}{P+N}\right]}_{k}\times 100;$$

**Figure 10:**
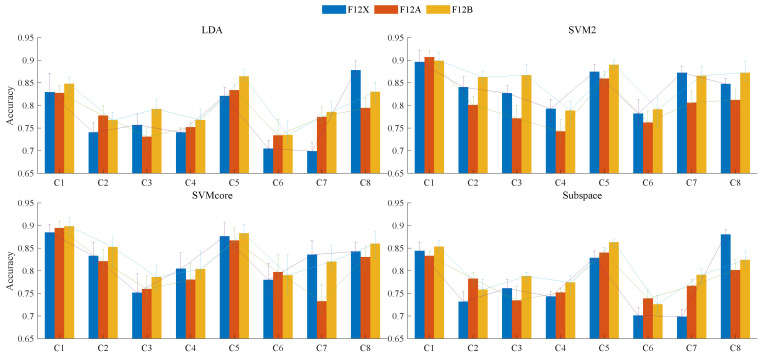
Classification results comparison between 3 feature groups of size 12.

Figure 10 displays the classification results, including the mean accuracy and error, for models trained with the four machine learning methods across three feature combinations on eight sEMG channels. The mean of the 20 replicates ensured the reproducibility of these experimental results. Among the four methods, the F12B feature group exhibited superior performance compared to F12A and F12x, yielding the highest average accuracy with relatively small variations across channels (as shown by the line chart amplitude fluctuations for each group). F12X achieved relatively high accuracy in identifying C8 channel signals using the LDA method, but its accuracy fluctuated significantly across channels, resulting in poorer overall performance. Overall, we selected the F12B feature group as the key parameter to differentiate the upper arm muscle groups of B-PE majors with long-term exercise from those of regular students.

**Figure 11:**
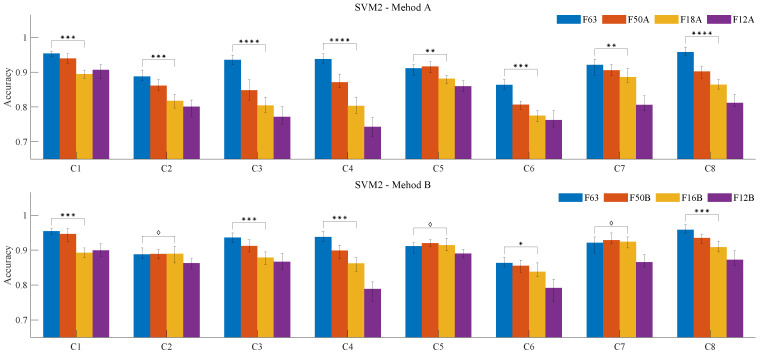
Comparison and significance analysis of classification results for different feature groups. ♢ $p>=5\times 10^{-2}$, * $p<5\times 10^{-2}$, ** $p<1\times 10^{-10}$, *** $p<1\times 10^{-20}$, **** $p<1\times 10^{-30}$.

To further compare the effectiveness of feature extraction Methods A and B, we trained the SVM2 model to evaluate the classification performance of the feature groups generated at each stage of both methods. As shown in Figure 11, F63 represents the classification results using all features, and serves as the control group for both the upper and lower sections of the figure. The feature groups F50A, F18A, and F12A were produced through the three-stage process of Method A, while F50B, F16B, and F12B were generated similarly by Method B. We observed that certain channels—specifically, C1, C3, C4, and C8—showed higher recognition accuracy when using all 63 features. Among these, C1 and C3 correspond to the biceps brachii of both arms, C4 represents the triceps brachii muscle of the left arm, and C8 represents the flexor carpi muscle of the right arm. A previous study by Triwiyanto et al. [47] suggested that EMG signals from the triceps generally exhibit lower activity than those from the biceps. Our results support this finding in terms of recognition accuracy yet also reveal that long-term exercise affects not only the biceps but also the triceps brachii of the non-dominant arm and the flexor carpi of the dominant hand. This is particularly evident since all participants were right-handed, suggesting that prolonged exercise can significantly influence muscles on both dominant and non-dominant sides.

As shown in Figure 11, the model accuracy of Method B decreased more gradually across the three stages. We performed a quantitative analysis of the significant differences in classification accuracy between feature groups F18A and F16B, generated in the second step of each method, and feature group F63. The results are indicated in the figure; a diamond shape indicates no significant difference in the classification accuracy for different feature combinations based on a t-test at the 0.05 significance level, suggesting that certain feature groups, such as F63 and F16B, can be used interchangeably for channels C2, C5, and C7. More asterisks (*) represent a greater significance level, indicating a notable decrease in classification accuracy. Using F63 as the baseline, Method B typically showed a smaller reduction in accuracy compared to Method A, likely due to the exclusion of channel 6 features during training, which may have helped reduce noise in the samples. For a more intuitive expression of significance, we used the absolute decibel value of the *p*-value (see Equation (18)) as an indicator of classification accuracy differences between the target feature group and F63 in the trained SVM2 models. Higher values represent greater significant, while values <= 1.301 (*p* = 0.05) indicate no significant difference. As shown in Table 3, *x*-*y* represents a comparison between the *x*-th and *y*-th bars. Except for special channel and feature matching cases (such as [C1, 1-4]), Method B typically outperformed Method A in feature extraction across the various stages, with significant improvements in classification performance. The difference in classification accuracy caused by feature reduction was smaller.

(18) $$\left|dB\right|=\left|\log_{10}P-value\right|;$$

Based on the above analysis, balancing accuracy and efficiency, F12B emerges as the optimal feature combination. However, when higher recognition accuracy is required and more computational power is available, F16B serves as the best alternative. F12B includes nine TD features and three FD features: ASM, AR(4), DAMV, AAC, LTKEO, SSI, SD, WL, and DUR; and PKF, SM0, and Wprcoef(34). Compared to F12B, F16B adds four additional features: ASS, AR(2), CARD, and MNF, all of which are described in Section 2.3.2.

**Figure 12:**
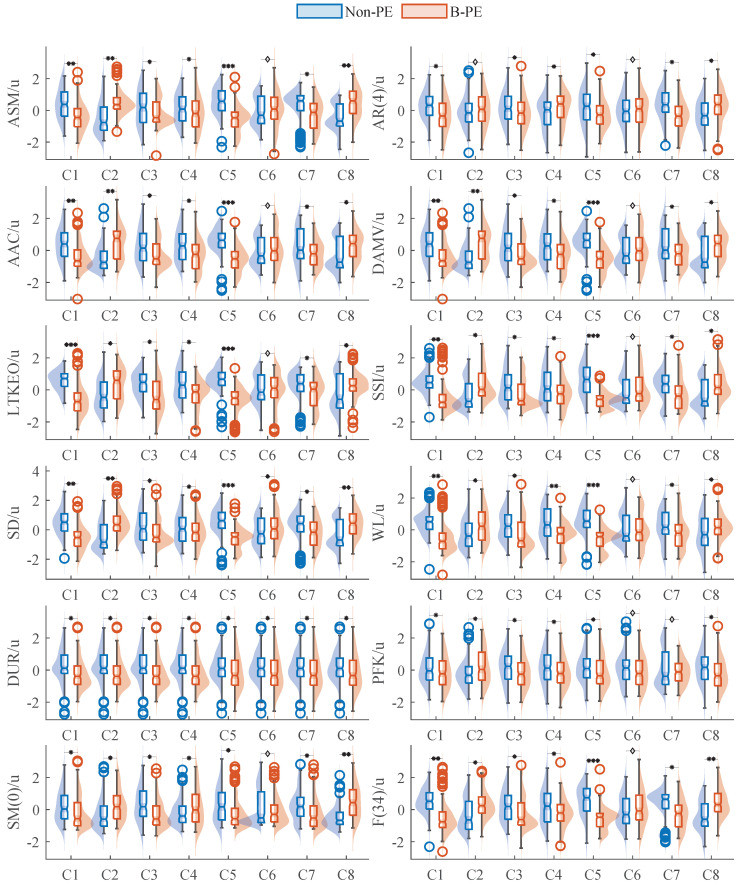
The distributions of features within F12B are compared between the B-PE group and the non-PE group. Here, u shows that the normalized feature is unitless. ♢ $p>=5\times 10^{-2}$, * $p<5\times 10^{-2}$, ** $p<1\times 10^{-10}$, *** $p<1\times 10^{-20}$.

Figure 12 presents the distribution of the 12 features from the event epoch EMG signals across eight channels for both amateur and professional subjects. Notches on the boxplots indicate whether the median values differ significantly. There are notable differences in the feature values for samples from the two groups in most cases. Light red and light blue filled areas represent the probability density function (pdf) of feature variables estimated by a kernel smoothing function [48]. The density estimates reveal a unimodal distribution for most features, while some display a bimodal pattern. Most features differ between amateurs and professionals in unimodal distributions, such as AR(4) features, DUR features, etc. Some features, like SSI in C2 or C8, show differences between groups in unimodal and bimodal distributions.

### 3.2. Influence Between Variables

We evaluated the performance of the F12B feature set across different channels, and the significance levels are indicated in Figure 12. Among the eight upper limb muscle groups analyzed, channels 1, 3, 4, 5, and 8 exhibited significant differences across all 12 features. Channels 2 and 7 displayed no significant differences in only one feature each; channel 2 showed no significant difference in the AR(4) feature, with similar feature distributions, while channel 7 lacked statistical significance in the PKF feature but exhibited some concentration differences in feature distribution. Notably, channel 6 showed significant differences in only two features, SD and DUR, in line with previous findings. This result may be related to the subjects’ right-handedness, which may limit training effects on the Flex. Carp muscle, as well as potential issues with signal quality. Overall, we found the F12B feature group identified by our proposed methodology to be broadly applicable across all eight upper arm muscle groups and useful as a generalized indicator for monitoring muscle training in male basketball players.

**Figure 13:**
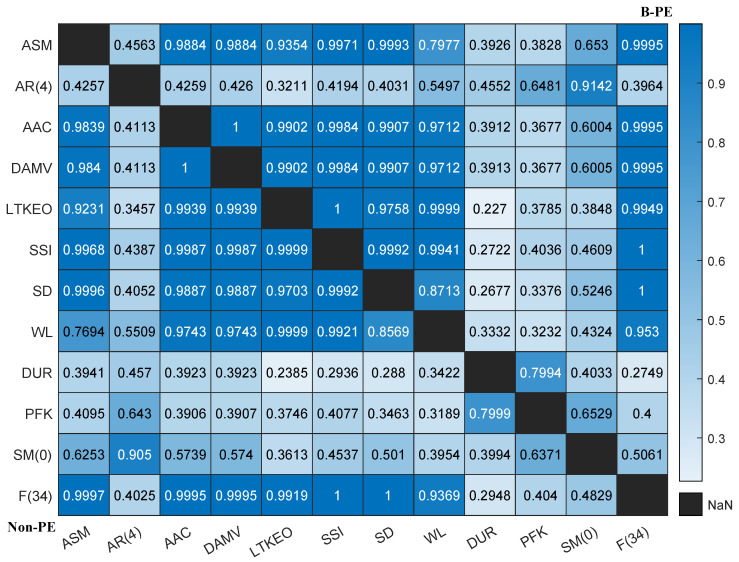
Heatmap of mean values from the Tukey HSD test. The color intensity represents the p-value, indicating the significance of the difference between each feature pair.

Additionally, we performed a statistical analysis to examine the effects among variables. For the F12B feature set, a one-way ANOVA was performed across the 12 features for specific subject groups and channels to determine *p*-values and assess significant differences between groups, as shown in Table 4. The results indicate no significant differences among the features for channels 3, 6, and 7. For the remaining five channels, where significant differences were found, post hoc analyses using the Tukey HSD test yielded pairwise p-values to clarify which specific groups exhibited statistically significant differences. Figure 13 illustrates the average significance of pairwise feature differences across these five channels. In the figure, the lower-left corner represents the non-PE group, the upper-right corner the B-PE group, and the diagonal shows the null value. Several time domain features—ASM, AAC, DAMV, LTKEO, SSI, SD, and WL—demonstrate strong correlations with one another. Features AR(4) and SM(0) show high mutual correlation but low correlation with other time domain features. Meanwhile, DUR, PKF, and SM(0) exhibit low correlation with other features. Notably, the wavelet packet decomposition feature F(34) is highly correlated with most time domain features, except AR(4). The results suggest a potential correlation between these features, showing consistent patterns for both the non-PE and B-PE groups.

## 4. Discussion

This paper focuses on identifying key EMG features and channels that are essential for monitoring muscle changes in male basketball players. EMG-based applications, such as gesture recognition [35], have shown promising results. Nazmi et al. [5] provide a comprehensive review of the methods for recognizing motion patterns during both isotonic and isometric contractions. However, few studies focus on the electromyographic differences in isotonic contractions between professional and amateur athletes. Compared to previous research, the accuracy results in Table 2 are not the highest reported. We believe this is due to two factors: first, the reported accuracy is the average of 20 repeated experiments performed for reproducibility rather than the optimal result from a single trial; second, EMG data were collected using a standardized setup across participants, without individual calibration. Compared to isometric exercises, features from isotonic exercises may more accurately capture athletes’ real-time performance. The findings of this paper offer valuable insights into using EMG features from isotonic exercises in sports training. Nonetheless, several limitations remain and warrant further investigation:

1. Unlike static posture analysis, such as gesture recognition, the isotonic contractions in the alternating curl experiments introduced greater signal noise, which may have impacted classification accuracy.
2. The study included only ten young male subjects, limiting generalizability and lacking insights from males of other ages and female subjects.
3. This study investigated the differences in sEMG features between professional and amateur students. For practical applications, a finer grading of athletic expertise would provide more detailed insights.

This study investigated the use of sEMG-based indicators to monitor the training effects on the upper arm muscles of male basketball players. By comparing various feature combinations, we identified the minimal set of features that efficiently captures muscle activity, offering a simplified yet effective approach for monitoring training effects. These findings have important implications for sports training and performance evaluation, including the following:

1. Simplification for practical use: Identifying a minimal feature set (such as the F12B feature group) makes it easier to implement sEMG-based muscle monitoring in real-world training settings.
2. Broad application across sports: Although this research focuses on male basketball players, the methodology and findings are applicable to a wide range of sports. The ability to monitor muscle performance with a minimal feature set can be adapted to other athletic contexts, such as strength training, rehabilitation, or cross-sport athlete monitoring.
3. In-depth training process supervision: The findings from this study provide a foundation for more refined monitoring of training phases and muscle adaptations. By using sEMG to track muscle activity during different phases of training, coaches can gain valuable insights into muscle fatigue, endurance, and recovery.
4. Potential for longitudinal tracking: Implementing minimal sEMG feature sets can also facilitate longitudinal tracking of muscle adaptation over time. This is especially valuable in sports where consistent muscle development is crucial, such as basketball, where upper body strength directly impacts performance. By regularly tracking changes in muscle activity and fatigue, athletes can adjust their training schedules to avoid both overtraining and undertraining, ultimately optimizing performance and minimizing the risk of injury.

Overall, the implications of this study extend beyond basketball training. By simplifying the sEMG-based muscle monitoring process and identifying key muscle performance indicators, this research contributes to more efficient and targeted sports training across a wide variety of athletic disciplines. It offers a pathway for more precise training supervision, enhancing the development of optimized, individualized training regimens and ultimately enhancing athletic performance.

## 5. Conclusions

This paper examines the effects of long-term, sustained basketball training on muscle development by comparing B-PE with non-PE students. An isotonic contraction exercise was designed to collect sEMG signals from the upper arm muscles of both groups. Two feature selection methods were proposed, resulting in six feature combinations: F50A, F18A, F12A, F50B, F16B, and F12B. By comparing three combinations with a feature size of 12—the control group F12X and groups F12A and F12B—we found that, across 20 rounds of ten-fold cross-validation using four machine learning methods, the F12B feature group achieved consistent accuracy ranging from 80% to 90% with the SVM2 model, outperforming other methods. The F16B feature group achieved even higher accuracy, ranging from 88% to 95%, making it ideal for scenarios where enhanced accuracy justifies the addition of four extra features. This study contributes to expanding the application of sEMG features in sports training. Future work will focus on exploring the specific features that change with exercise, aiming to better understand the underlying mechanisms. To monitor different stages of training progress, we also plan to collaborate with the PE department to categorize training effects more precisely. We will also collect a larger, more diverse set of experimental samples for deeper analysis.

## Figures and Tables

**Table 1:** Anthropometric information and average weekly exercise time of the subjects of the two groups.

	Age	Height/cm	Weight/kg	Exercise per Week/h
Amateurs	22	168	52.5	3
	24	168	60	6
	24	177	70	2.5
	26	184	90	5
	30	180	72	1.5
Professionals	18	175	52	30
	24	177	76	40
	27	183	91.5	36
	26	188	104	32
	31	183	88	45

**Table 2:** Mean and variance of classification accuracy.

		LDA	SVM2	SVMcore	Subspace
F12X	μ	0.771216	0.841602	0.826001	0.773535
	σ	0.064469	0.039990	0.045512	0.068727
F12A	μ	0.778101	0.807764	0.810278	0.781152
	σ	0.039180	0.053684	0.054118	0.040748
F12B	μ	0.798755	0.854590	0.836719	0.797241
	σ	0.044686	0.041740	0.042814	0.047032

**Table 3:** Significance comparison for each step in Methods A and B.

$\left|\mathrm{dB}\right|$	Method A			Method B		
	1-2	1-3	1-4	1-2	1-3	1-4
C1	8.626	27.408	20.561	2.569	28.596	24.639
C2	11.143	22.422	25.382	0.186	0.320	10.667
C3	24.721	30.934	35.955	11.141	22.103	20.392
C4	21.332	32.003	37.969	16.146	26.031	34.290
C5	0.972	11.971	19.051	2.312	0.447	8.312
C6	23.615	28.349	26.435	2.195	8.839	18.656
C7	5.037	11.796	29.083	1.732	0.377	18.671
C8	21.571	33.451	35.970	13.210	23.049	27.039

**Table 4:** The *p*-values from the one-way ANOVA of F12B features for each channel within the non-PE and B-PE groups.

*p*-Value	C1	C2	C3	C4	C5	C6	C7	C8
Non-PE	8.34 $\times \;10^{-5}$	6.87 $\times \;10^{-11}$	7.48 $\times \;10^{-1}$	5.66 $\times \;10^{-7}$	3.58 $\times \;10^{-9}$	2.90 $\times \;10^{-1}$	8.74 $\times \;10^{-1}$	1.00 $\times \;10^{-11}$
B-PE	5.08 $\times \;10^{-4}$	2.25 $\times \;10^{-10}$	6.66 $\times \;10^{-1}$	1.51 $\times \;10^{-7}$	9.27 $\times \;10^{-13}$	4.71 $\times \;10^{-1}$	7.56 $\times \;10^{-1}$	1.01 $\times \;10^{-13}$

## Data Availability

The preprocessed data of this study are available from the first author upon reasonable request submitted to [2110108@stu.neu.edu.cn].

## Abbreviations

The following abbreviations are used in this manuscript:

sEMG	Surface electromyography
B-PE	Basketball physical education
TD	Time domain
FD	Frequency domain
ML	Machine learning
Flex./Ext. Carp.U	Flexor/extensor muscle groups
ICC	Intraclass correlation coefficient
SVM	Support Vector Machine
LDA	Linear Discriminant Analysis

## Appendix A

A	Feature extraction utilizing all 8 channels
1.	Find a ID set $X_{1}$ of Fx, if $\sum_{C1}^{C8}P_{Fx}>1$;
	Find a ID set *Y* of Fx, if $\sum_{C1}^{C8}H_{Fx}<6$;
	$Z_{1}=X\cup Y;$
	>>$Z_{1}$ = [2, 3, 4, 5, 6, 10, 18, 19, 29, 34, 37, 47, 56];
2.	Find $Z_{2}$ = [1, 8, 9, 14, 15, 17, 25, 31, 32, 35, 36, 40, 44, 45, 48, 49, 51, 60];
3.	Find a ID set $X_{0.5}$ of Fx, if $\sum_{C1}^{C8}P_{Fx}>0.5$;
	$Z_{3}=X_{0.5}\cap Z_{2};\;>>$$Z_{3}$ = [9, 15, 31, 35, 45, 49];

B	Feature extraction utilizing the 7 channels without C6
0.	Set $HR_{Fx}\left[CR\right]\;includes\;H_{Fx}\left[C\right];$
	Set $PR_{Fx}\left[CR\right]\;includes\;P_{FCx}\left[C\right];$
	CR⊂[1,2,3,4,5,6,7],C⊂[1,2,3,4,5,7,8];
1.	Find $Z_{1}$, if $\sum_{CR1}^{CR7}HR_{Fx}<6$;
	>>$Z_{1}$ = [3, 5, 10, 18, 19, 31, 34, 35, 37, 45, 47, 49, 56];
2.	Find $Z_{2}$ = [1 2 4 6 7 9 11 23 33 36 41 44 46 48 51 60];
3.	Find a ID set $X_{0.4}$ of Fx, if $\sum_{CR1}^{CR7}PR_{Fx}>0.4$;
	$Z_{3}=X_{0.4}\cap Z_{2};\;>>$$Z_{3}$ = [2, 4, 9, 46];
